# Human Leukocyte Antigen (*HLA*) Signatures and Idiosyncratic Drug-Induced Liver Injury

**DOI:** 10.3390/ijms27010482

**Published:** 2026-01-02

**Authors:** Alexia Onaciu, Alina Grama, Ștefan Agoșton, Alexandra Mititelu, Bianca Mariş, Horia Ştefănescu, Tudor Lucian Pop

**Affiliations:** 1 Faculty of Medicine, “Iuliu Hațieganu” University of Medicine and Pharmacy, 400012 Cluj-Napoca, Romania; 2 2nd Pediatric Discipline, Department of Mother and Child, Faculty of Medicine, “Iuliu Hatieganu” University of Medicine and Pharmacy, 400012 Cluj-Napoca, Romania; 3 2nd Pediatric Clinic, Emergency Clinical Hospital for Children, 400370 Cluj-Napoca, Romania; 4 “Prof. Dr. Octavian Fodor” Regional Institute of Gastroenterology and Hepatology, 400162 Cluj-Napoca, Romania; 5 Department of Internal Medicine, “Iuliu Hațieganu” University of Medicine and Pharmacy, 400012 Cluj-Napoca, Romania

**Keywords:** drug-induced liver injury, *HLA* polymorphisms, adaptive immune response, idiosyncratic drug reactions

## Abstract

Drug-induced liver injury (DILI) remains one of the most challenging adverse drug reactions in clinical practice, particularly in its idiosyncratic form, which is not dose-dependent and is largely driven by host-specific immune and genetic factors. Recent genomic studies have revealed strong associations between certain human leukocyte antigen (*HLA*) alleles and susceptibility to DILI, supporting an immunogenetic mechanism in which drug or metabolite–protein adducts act as neoantigens, triggering aberrant T-cell activation and hepatocellular injury. This review summarizes current evidence on the contribution of *HLA* polymorphisms to the pathogenesis of idiosyncratic DILI, highlighting allele-specific risk patterns, such as *HLA*-B*57:01 associated with flucloxacillin, *HLA*-DRB1*15:01–DQB1*06:02 in amoxicillin–clavulanate, and HLA-B*35:02 in minocycline-induced liver injury. Furthermore, ethnic variability and allele-haplotype interactions are discussed as potential modulators of susceptibility and clinical phenotype. By integrating genetic and immunological insights, the identification of *HLA* signatures offers promising tools for precision medicine, enabling earlier identification of at-risk individuals and improved prevention of severe hepatotoxic reactions.

## 1. Introduction

Drug-Induced Liver Injury (DILI) is an acute or chronic liver injury secondary to exposure to certain xenobiotics, mainly pharmaceutical drugs. Depending on the type of toxic agent, hepatotoxic reactions can be classified as either intrinsic or idiosyncratic. Intrinsic hepatotoxicity is a dose-dependent toxicity that appears shortly after exposure, due to exceeding the liver detoxification capacity by administering overdoses. Histological findings are characterized by hepatic necrosis or steatosis, which may result from the direct action of the chemical substance or its metabolites (direct hepatotoxicity) or from disruption of intracellular metabolic processes (indirect toxicity). Acetaminophen is the prototype agent of direct hepatotoxicity, whereas the toxicity of Amanita phalloides is often indirect, by inhibiting RNA polymerase II and impairing protein synthesis [1,2]. The liver functions as the primary organ of biotransformation, converting lipophilic xenobiotics into hydrophilic metabolites mainly through cytochrome P450–mediated oxidative pathways, thereby facilitating their excretion via renal or biliary routes [3]. Mechanisms of hepatotoxicity predominantly involve the hepatic biotransformation of drugs into reactive metabolites, which may exceed the liver’s intrinsic detoxification and antioxidant capacities. These metabolites can cause hepatocyte necrosis through oxidative stress or mitochondrial dysfunction and activation of intracellular signaling networks and programmed cell death [4]. These reactive intermediates trigger an immunologically mediated response by initiating immune dysregulation, loss of self-tolerance, and the formation of neoantigens [5].

Idiosyncratic DILI is a condition in which liver damage occurs because of exposure to pharmaceutical drugs, herbal products, or other xenobiotics, independent of dose. Usually, toxicity results from individual genetic factors, immune-mediated mechanisms, or metabolic variations that influence a patient’s susceptibility to hepatotoxicity [1]. Host-specific immune factors, particularly specific human leukocyte antigen (*HLA*) haplotypes, are involved in this relation between genetic susceptibility and aberrant immune activation, highlighting the complexity of idiosyncratic DILI and contributing to the ongoing challenges in its early diagnosis and prevention [1,6]. In idiosyncratic toxicity, genetic factors play a significant role. Mutations in genes encoding enzymes involved in Phase I and Phase II hepatic metabolism increase susceptibility to toxic liver injury [5,7]. The best example of metabolic idiosyncratic hepatotoxicity is isoniazid, whose toxicity is influenced by genetic mutations that affect the activity of N-acetyltransferase 2, the enzyme responsible for its hepatic metabolism.

Both types of DILI, intrinsic and idiosyncratic, share common molecular features, including disruption of calcium homeostasis, damage to cellular or canalicular membranes, metabolic bioactivation, triggering of autoimmunity, and mitochondrial impairment, leading to hepatocyte apoptosis and necrosis [3].

Multiple host-related factors (including age, genetic predisposition, and autoimmune activation) and environmental factors (exposure to industrial chemicals such as carbon tetrachloride, toxins such as aflatoxin, and pollutants) can trigger DILI [8]. Although advanced age is generally associated with an increased susceptibility to hepatotoxicity, children may experience more severe liver injury after exposure to specific xenobiotics such as valproic acid or aspirin. This increased vulnerability in the pediatric population may be attributed to immature hepatic metabolic capacity. The result is reduced expression of drug transport proteins and increased accumulation of toxic intermediates, exacerbating hepatocellular damage [1,2,6].

The major histocompatibility complex (MHC) consists of a group of genes located on the short arm of chromosome 6 that encode certain membrane glycoproteins. There are three known classes of *HLA*: Class I (*HLA-A*, *-B*, and *-C*), Class II (*HLA-DR*, *-DP*, *-DQ*), and Class III (including tumor necrosis factor, some cytokines, interleukins, and complement components). Children’s predisposition to develop autoimmune hepatitis (AIH) is associated with Class II *HLA* genes, specifically the DRB1 locus. This gene encodes proteins involved in the immune system that distinguish self-proteins from non-self proteins, such as proteins of bacterial or viral origin [5]. *HLA* molecules play a critical role in the host immune response by presenting non-self antigens to CD4^+^ T helper lymphocytes (Th1 and Th2), thereby eliciting both humoral and cell-mediated immune responses [5].

Current evidence supports the involvement of the MHC in the initiation of idiopathic AIH. Specifically, the alleles HLA-DRB1*03:01, DRB3*01:01, and DRB1*04:01, which encode the *HLA-DR3*, *DR52*, and *DR4* molecules, respectively, are well known for their role in triggering type 1 AIH in European and North American populations. Furthermore, the role of certain drugs in triggering autoimmune diseases is also recognized, as they may induce latent immunity [9,10]. The presence of *HLA*-DRB1*03:01 or *04:01* alleles supports the diagnosis of idiopathic AIH, whereas the detection of DILI-associated alleles favors drug-induced AIH (DI-AIH). Notably, *HLA*-DRB1*15:01, a known DILI risk allele, is less frequently observed in AIH than in healthy individuals, underscoring the utility of genetic testing to distinguish between these conditions [11]. Associations have also been identified between specific *HLA* class II alleles and the pattern of liver injury in drug-induced hepatotoxicity. In particular, the alleles *HLA*-DRB1*15 and *HLA*-DQB1*06 have been linked to cholestatic features, whereas *HLA*-DRB1*07 and *HLA*-DQB1*02 appear to confer a protective effect. Nevertheless, not all individuals carrying these alleles develop hepatotoxicity, suggesting that additional host, metabolic, or environmental factors contribute to disease susceptibility [12].

This narrative review analyses current evidence on *HLA* involvement in the genetic predisposition to DILI, providing a preliminary understanding of a potential key tool for advancing predictive and precision medicine in hepatology.

## 2. Correlations Between DILI and AIH

In genetically predisposed individuals, the immune response against hepatic structures may be initiated following viral infection or exposure to certain drugs or toxic agents. These exposures can promote the formation of drug–protein adducts (neoantigens), which determine autoimmune-like reactions leading to DI-AIH or immune-mediated liver injury. This immune-mediated process involves B cell activation and the production of autoantibodies, which initiate hepatic parenchymal inflammatory cascades, leading to portal and periportal inflammation, interface hepatitis, and, in severe cases, hepatocellular necrosis [13]. The processes share many clinical and histopathological features with idiopathic AIH, making differential diagnosis difficult [13].

After a xenobiotic enters the body, the liver’s metabolic system converts it into non-toxic, water-soluble metabolites, which are then eliminated. In some cases, this bioactivation process generates reactive intermediates that covalently bind to cellular macromolecules (such as proteins and lipids), forming drug–protein adducts (hapten–carrier complexes). These modified structures are recognized by the immune system as neoantigens, causing immune-mediated reactions. The responses are classical hypersensitivity reactions, directed against the xenobiotic itself, or autoimmune-like reactions, in which hepatocytes become unintended targets of the adaptive immune response [14]. An example is halothane-induced hepatotoxicity, which represents a type II hypersensitivity reaction [5]. During halothane metabolism via cytochrome P450 2E1 (CYP2E1), reactive intermediates, such as trifluoroacetylated species, are generated, which covalently bind to hepatic proteins, forming neoantigenic complexes. It will trigger the production of anti-trifluoroacetyl and anti-CYP2E1 autoantibodies against hepatocytes, leading to complement activation, cellular cytotoxicity, and hepatic necroinflammation [15,16].

Correlations between hepatic toxicity and AIH are well established. Drugs can cause toxic hepatitis with autoimmune features or may act as triggers for AIH [17]. Both conditions can present similar clinical, laboratory, and histological features. Differentiating idiosyncratic hepatotoxicity with autoimmune features from true AIH represents a real challenge for clinicians, especially when specific antibodies are not detected or immunoglobulin G levels remain normal. Liver biopsy and histological examination could distinguish between these two conditions. The presence of interface hepatitis, periportal inflammation, or emperipolesis is suggestive of idiopathic AIH. In contrast, toxicity is typically associated with an increased number of neutrophils in the portal vein spaces [17]. Three main forms of immune-mediated drug-induced liver pathology (DI-AILD) are currently described: autoimmune hepatitis with overlapping drug-induced liver injury (AIH with DILI), drug-induced autoimmune hepatitis (DI-AIH), and immune-mediated DILI (IM-DILI) [10]. Distinguishing among these is important, especially since AIH typically requires long-term immunosuppressive therapy. In children, this approach carries potential risks, not only for growth and development but also for social and cognitive well-being. In contrast, idiosyncratic DILI generally does not demand such intensive treatment [10]. Before confirming a diagnosis of idiopathic AIH triggered by drugs or identifying a specific drug as the cause of IM-DILI, genetic predisposition and *HLA*-*DR* class II typing could be useful. Although the MHC is thought to contribute to autoimmune liver responses, its role in drug-induced toxicity is less defined. Also, DILI itself may act as a trigger for AIH in susceptible individuals. *HLA*-DRB1*03:01 and *HLA*-DRB1*04:01 alleles, which represent well-established genetic risk factors for idiopathic AIH, do not significantly increase susceptibility to DI-AIH. This finding suggests that DI-AIH and classical AIH, while sharing immunopathological features, may involve distinct genetic and mechanistic pathways [18].

## 3. Present Evidence on *HLA* Associations with DILI (Table 1)

### 3.1. Non-Steroidal Anti-Inflammatory Drug (NSAIDs)

Immunologic DILI is an immune-mediated hypersensitivity reaction to a specific xenobiotic that predominantly occurs in people with an inadequate adaptive immune response. This type of toxicity depends on the host’s immune system, including *HLA*, as well as the chemical properties of the xenobiotic, its metabolites, or reactive haptens [19,20]. Often, this type of hepatotoxicity is accompanied by extrahepatic symptoms, such as skin rashes and eosinophilia, and its severity is directly related to the individual’s immune tolerance. In a study by Bonkovsky HL, diclofenac was the most frequently implicated agent in NSAID-induced hepatotoxicity, accounting for more than 90%, and ibuprofen accounted for less than 10%. For both drugs, a close association with genetic predisposition has been identified. HLA-DRB1*04:03 increased the hepatotoxicity risk of those drugs by eightfold and has been associated with a severe phenotype, often co-occurring with HLA-DQA1*03:01. These associations were consistent across populations and predominantly driven by acetic acid derivative NSAIDs [21]. In addition, another allele, HLA-B*35:03, was linked to a higher risk of NSAID-related liver injury, mainly in non-Hispanic White and Hispanic populations, where it is much more common than in non-Hispanic Black individuals [21].

In contrast, the HLA-A*02:01 allele was associated with a significant, independent protective effect against NSAID-induced liver injury, most evident in cases involving diclofenac. These findings support a dual model of genetic susceptibility and protection, in which HLA-DRB1*04:03 predisposes and HLA-A*02:01 protects against NSAID-induced hepatotoxicity [21]. Even though ibuprofen is a widely prescribed, over-the-counter drug, it has been generally thought to be safe regarding liver toxicity. However, cases of ibuprofen-induced DILI arise, while the Spanish and Latin American DILI registries found higher than expected rates [22]. Reports of *HLA* susceptibility alleles for ibuprofen-induced DILI are scarce, but Gui et al. identified HLA-B*58:01 in a pediatric patient who developed Stevens-Johnson syndrome and liver injury following ibuprofen treatment [23].

### 3.2. Nitrofurans and Tetracyclins

The potential of nitrofurans and some tetracyclines to induce AIH in genetically susceptible individuals is already known. A high titer of antinuclear autoantibodies (ANA) was found in nitrofurantoin (52%) and minocycline (57%) DILI cases [24]. The HLA-B*35:02 allele has been identified as a potential genetic risk factor for minocycline-induced DILI [25]. This association, first detected through genome-wide association studies (GWAS) and subsequently confirmed by sequence-based *HLA* typing, suggests that drug-specific genetic susceptibility to DILI may involve both Class I and Class II *HLA* alleles. The HLA-B*35:02 allele is rare in the general population, with an estimated frequency of approximately 0.3% in Caucasians and less than 0.1% in African Americans. Importantly, this relationship does not appear to reflect confounding by indication, as previous GWAS in acne patients (the main population receiving minocycline) have shown no association between this allele and acne [25]. Affected individuals present with autoimmune-like features (including positive autoantibody profiles and hypergammaglobulinemia, at the time of diagnosis) and liver biopsy findings often reveal interface hepatitis and plasma cell infiltration, closely resembling AIH. Approximately half of the patients received corticosteroid therapy with liver injury remission, 24% progressed to chronic DILI, with no cases with liver transplantation or fatal evolution. This suggests that HLA-B*35:02 may predispose to a slow evolution, towards chronicity [25].

Nitrofurantoin-induced liver injury may occur after only a few days of treatment, but it is more commonly seen after prolonged use, more than one year. Nitrofurantoin hepatotoxicity is associated with the HLA-DRB1*11:04 allele, which was observed approximately 4 times more frequently in American patients of European descent who developed hepatotoxicity than in the control population [26]. This allele was present exclusively in individuals who had long-term exposure to nitrofurantoin, suggesting a potential exposure-dependent genetic predisposition. Chronic exposure is frequently associated with autoimmune-like features and histological evidence of chronic hepatitis and fibrosis, often necessitating corticosteroid therapy. Long-term users of nitrofurantoin are therefore at risk of significant morbidity and mortality, and regular monitoring of ALT every 3–6 months is recommended in patients receiving extended therapy [26]. In contrast, cases of liver injury following short-term nitrofurantoin therapy showed a higher prevalence of the extended haplotype HLA-A*01:01–B*08:01–C*07:01. These *HLA* markers, which correlated with treatment duration, could serve as markers to identify patients at risk of nitrofurantoin hepatotoxicity [26].

Analysis of data from the Drug-Induced Liver Injury Network (DILIN) demonstrated that most cases of DILI associated with nitrofurantoin or minocycline, and approximately half of those linked to methyldopa or hydralazine, exhibited autoimmune features resembling AIH. However, these autoimmune manifestations generally diminished as hepatic injury resolved and were not associated with the classical *HLA* alleles known to confer susceptibility to AIH [27].

### 3.3. Flucloxacillin

The increased risk of hepatotoxicity in some patients after taking certain medications may be explained by the preferential molecular association of specific drugs or their metabolites with various peptides that bind *HLA* molecules. A good example is flucloxacillin, which is associated with HLA-B*57:01, or amoxicillin with HLA-DRB1*06:02 [28].

Flucloxacillin is a common cause of DILI, especially in Northern Europe and Australasia, with incidence rates increasing based on the number of prescriptions and patient age. While most patients recover completely, some may develop chronic liver conditions such as vanishing bile duct syndrome, and in rare cases, a fatal outcome. Initially thought to be caused by impaired drug metabolism, flucloxacillin DILI often presents with hypersensitivity features like rash and fever. The HLA-B*57:01 gene plays a key role in flucloxacillin-induced DILI; 85% of patients with this condition carry this allele. CD8-positive T cells from individuals with this allele can be activated by flucloxacillin via drug-protein conjugates, though non-hapten pathways have also been proposed [28]. The allele HLA-B*57:03, which is structurally related to HLA-B*57:01, has also been associated with an increased risk of flucloxacillin-induced DILI. At the molecular level, the presence of valine at position 97, a residue shared by both HLA-B*57:01 and HLA-B*57:03, appears to be a key determinant of susceptibility, likely influencing the conformation of the peptide-binding groove and the presentation of flucloxacillin-modified self-peptides to cytotoxic T cells [29].

CD4^+^ and CD8^+^ T cells isolated from individuals who developed flucloxacillin-induced DILI were shown to mount antigen-specific immune responses following presentation of flucloxacillin-derived peptides by dendritic cells. Naïve CD8^+^ T cells from HLA-B*57:01-positive individuals became activated upon exposure to these antigens and expressed chemokine receptors CCR4 and CCR9, enabling migration toward the liver in response to CCL17 and CCL25. The activated T-cell clones secreted interferon-γ (IFN-γ), Th2 cytokines, and cytotoxic mediators, including perforin, granzyme B, and Fas ligand (FasL), which collectively contribute to hepatocellular injury [29].

Flucloxacillin was shown to form covalent adducts with lysine residues on serum albumin in a time-dependent manner, and the extent of this binding correlated directly with the magnitude of T-cell activation. The activation of CD8^+^ T cells required antigen processing. It was restricted by the presence of HLA-B*57:01 and the closely related HLA-B*58:01 alleles, underscoring the critical role of antigen presentation and MHC class I specificity in the pathogenesis of this immune-mediated form of DILI [29].

### 3.4. Amoxicillin-Clavulanate

The role of *HLA* polymorphisms is particularly evident in amoxicillin–clavulanate-induced liver injury, one of the most frequent causes of idiosyncratic DILI in Western countries. Hautekeete et al. reported that 57% of patients with amoxicillin–clavulanate-associated hepatitis carried the HLA-DRB1*15:01 allele, compared with only 12% of healthy controls, supporting a strong immunogenetic predisposition. However, subsequent studies have shown that *HLA* genotype alone does not fully predict hepatotoxic risk, reinforcing the multifactorial nature of DILI pathogenesis [30,31].

Donaldson et al. conducted a large, population-based study to define further the *HLA* associations with amoxicillin–clavulanate-induced DILI. Two major findings emerged. First, the study confirmed the previously reported association between the HLA-DRB1*15:01–DQB1*06:02 haplotype and increased susceptibility. Individuals carrying the DRB1*15:01 allele were significantly over-represented among DILI cases [31]. Second, a protective effect was identified for the HLA-DRB1*07 allele family, which was markedly under-represented among affected patients [31]. As anticipated, all individuals carrying DRB1*15:01 also possessed the DQB1*06:02 allele, consistent with strong disequilibrium linkage between these loci. Collectively, these findings confirm a risk-protective dichotomy between DRB1*15:01–DQB1*06:02 susceptibility and HLA-DRB1*07 protection in the genetic predisposition to co-amoxiclav hepatotoxicity [31].

Hautekeete et al. identified a significant association between the HLA-DRB1*15:01–DRB5*01:01–DQB1*06:02 haplotype and immunoallergic hepatitis induced by amoxicillin–clavulanate. Through DNA-based *HLA* typing, this haplotype was shown to confer a markedly increased susceptibility to drug-induced liver injury [32]. Comparison between carriers and non-carriers revealed a distinct pattern of hepatic injury: individuals harboring the haplotype more frequently exhibited cholestatic or mixed forms of hepatitis, whereas none presented with a purely hepatocellular pattern. Other clinical, biochemical, and histopathological characteristics did not differ significantly between the groups. Notably, all patients who displayed extrahepatic hypersensitivity manifestations, including interstitial nephritis, cutaneous rash, or arthritis, and one individual with marked eosinophilia, carried the DRB1*15:01–DRB5*01:01–DQB1*06:02 haplotype. These findings support a model in which immune-mediated mechanisms, influenced by *HLA* class II genotype, play a central role in amoxicillin–clavulanate–associated hepatotoxicity [32].

### 3.5. Trimethoprim-Sulfamethoxazole

Trimethoprim–sulfamethoxazole (TMP–SMX)-induced DILI has been linked to distinct *HLA* class I alleles across different ethnic populations. Li et al. reported that HLA-B*14:01 was significantly associated with TMP-SMX-related hepatotoxicity in European Americans, with carriers exhibiting a 9.2-fold higher risk of liver injury than non-carriers. The allele frequency of HLA-B*14:01 was approximately 5.5 times greater in affected European American patients than in ethnically matched controls [33]. In contrast, within the African American subgroup, HLA-B*35:01 emerged as the principal genetic factor potentially conferring susceptibility to TMP–SMX-induced hepatotoxicity. Although the cohort comprised only 10 African American cases, 50% of affected individuals carried the *HLA*-B*35:01 allele, a markedly higher frequency than observed in the control population. These findings underscore the ethnic diversity of *HLA*-associated genetic risk for TMP–SMX hepatotoxicity and highlight the need for population-specific pharmacogenetic screening to improve the prediction and prevention of idiosyncratic DILI [33].

### 3.6. Macrolides

Azithromycin-induced drug-induced liver injury (AZ-DILI) has been associated with the *HLA* class II allele HLA-DQA1*03:01, which appears to act as a genetic susceptibility factor for this adverse reaction. The allele was identified at approximately 2.6 times the frequency in AZ-DILI patients compared to the general population [34]. Among non-Hispanic white individuals, carriers of DQA1*03:01 seemed to have a lower likelihood of developing chronic liver injury than non-carriers, although this trend requires further confirmation. No association was observed between DQA1*03:01 and amoxicillin–clavulanate-induced DILI (AC-DILI), suggesting that this genetic marker is specific to azithromycin-related hepatotoxicity [34]. Further haplotype analysis revealed that the DQ8 haplotype, composed of HLA-DQA1*03:01–DQB1*03:02, showed an even stronger correlation with AZ-DILI than HLA-DQA1*03:01 alone. This finding indicates that the DQ8 haplotype may serve as a more precise and reliable genetic marker for identifying individuals at increased risk of AZ-DILI, supporting its potential use in pharmacogenetic screening strategies [34].

Other macrolides have also been involved in DILI pathogenesis. Most evidence points towards erythromycin, with a relative risk (RR) of 3.7 [35]. The mechanism by which erythromycin causes DILI is different than that of other macrolides, by bile acid accumulation [36]. Some *HLA* alleles were found to be associated with erythromycin-induced DILI, namely HLA-A*33:01 and HLA-A*33:03, but these are not specific, as they have also been reported for enalapril and sertraline [37].

### 3.7. Fluoroquinolones

Fluoroquinolones are broad-spectrum antibiotics commonly used in adult infections but are limited in pediatrics due to age-specific adverse reactions and are used to treat multidrug-resistant infections [38]. DILI can occur with any agent in this class, with no clinical differences among ciprofloxacin, levofloxacin, and moxifloxacin, in correlation with HLA-DQA1*03:01 and HLA-B*57:01, with a high incidence of combined carriage [39].

### 3.8. Antituberculosis Drugs

Although antituberculosis DILI (ATLI) has been widely studied, no strong, consistent *HLA* associations have been identified. However, Nicoletti et al. reported suggestive evidence that a rare haplotype, *HLA-C12:02–B52:01*, may confer increased susceptibility in certain individuals [40]. This haplotype was previously associated with Crohn’s disease in Asian populations. HLA-B*52:01 allele demonstrated a stronger individual association than HLA-C*12:02, implying that the *HLA*-*B* locus may play a primary role in mediating this risk. Although only a small number of patients carried the haplotype, its reproducibility across cohorts and known autoimmune associations support its potential relevance. Interestingly, this association was absent in patients receiving isoniazid monotherapy, suggesting that the signal may reflect hepatotoxicity induced by another anti-TB drug, such as pyrazinamide [3,40]. Furthermore, while an earlier *HLA* class II typing study in an Indian population reported that the absence of HLA-DQA1*01:02 and the presence of HLA-DQB1*02:01 increased the risk of anti-TB DILI, Nicoletti et al. found elevated frequencies of *HLA*-DQA1*01:03 and HLA-DQA1*03:01 in their cohort—though these associations were not replicated in European patients, indicating possible ethnic or drug-specific variability in genetic susceptibility [40]. Chen et al. investigated the potential association between *HLA-DQB1* polymorphisms and the risk of ATLI. No statistically significant differences were initially observed between cases and matched controls [41]. However, individuals homozygous for HLA-DQB1*05 appeared more frequently among patients who developed ATLI than in the control group. After adjusting for potential confounding factors, including body weight and the use of hepatoprotective agents, multivariate analysis demonstrated a significant difference in the distribution of the HLA-DQB1*05/05 genotype between groups [41]. The odds ratio associated with this genotype remained consistently elevated across all statistical models, suggesting a potential true genetic association between *HLA-DQB1* variants and susceptibility to ATLI. These findings point to a possible role of HLA-DQB1*05 homozygosity in predisposing certain individuals to anti-TB drug-related hepatotoxicity, although further validation in larger, ethnically diverse cohorts is warranted [41].

### 3.9. Carbamazepine

The HLA-A*31:01 allele has recently been investigated as a potential genetic risk factor for carbamazepine-induced DILI. Among all DILI cases analyzed, 33% of patients carried this allele, corresponding to an odds ratio (OR) of 7, which exceeds previously reported associations involving class II *HLA* alleles linked to lumiracoxib-induced DILI [42]. Both carbamazepine-induced severe cutaneous adverse reactions and carbamazepine-related DILI share a unique amino acid residue (isoleucine at position 73) that defines a cryptic epitope specific to *HLA*-*A31* and *HLA*-*A33* molecules. Under physiological conditions, this epitope is normally concealed within the *HLA* structure; however, conformational changes in the antigen–peptide complex, such as dissociation of β_2_-microglobulin, may expose this residue and trigger an autoimmune response. HLA-A*33:01 and HLA-A*33:03 have been associated with DILI from several unrelated agents, suggesting a broader pattern of shared, immune-mediated susceptibility across different drugs. Nonetheless, in this study, the association with HLA-A*31:01 did not achieve genome-wide significance, most likely due to the limited sample size [42]. The authors propose that distinct mechanisms of antigen presentation may operate in the skin and liver, reflecting the liver’s central role in drug metabolism and the generation of reactive intermediates that can influence organ-specific immune activation [42].

### 3.10. Allopurinol

In allopurinol-induced liver injury, HLA-B*58:01 remains the most consistently identified risk allele, particularly among African and Asian populations. Recent data also suggest novel associations involving HLA-B*53:01 and HLA-A*34:02, which were observed more frequently in African American patients. Haplotype analysis indicates that the effect of HLA-B*53:01 may derive from its linkage disequilibrium with HLA-A*34:02, whereas HLA-B*58:01 appears to act independently [43]. These results not only confirm the established role of HLA-B*58:01 in allopurinol liver involvement and cutaneous hypersensitivity but also highlight the possibility of ethnic-specific susceptibility haplotypes contributing to immune-mediated DILI. These observations underscore the critical role of *HLA* class I molecules in shaping drug-specific and tissue-selective immune responses, underscoring the need for integrated pharmacogenetic screening in high-risk populations [43].

### 3.11. Ticlopinide

HLA-A*33:03 has been identified as a significant genetic risk factor for ticlopidine-induced DILI, particularly the severe cholestatic form, in Japanese individuals. This adverse reaction may be associated with the allele itself or with a specific haplotype encompassing *HLA-A33:03*, -*B44:03, -C14:03*, -DRB1*13:02, and -DQB1*06:04. Given the central role of *HLA* molecules in antigen presentation and immune activation, the observed association strongly supports an immune-mediated pathogenesis, which could explain the higher incidence of ticlopidine-induced liver injury reported in Japanese patients [44]. Nicoletti et al. also reported a strong association between HLA-A*33:03 and cholestatic DILI. HLA-A*33:03 is relatively prevalent in East Asian populations, with an estimated carrier frequency of 10–15%, which may partially account for the ethnic variability observed in susceptibility to ticlopidine-induced hepatotoxicity [44].

### 3.12. Antiepileptic Drugs

*HLA*-B*53:01 has emerged as a potential genetic risk factor for aromatic antiepileptic drug (AED)-induced liver injury, with the strongest association observed in phenytoin-related cases [44,45]. Children appear to be particularly vulnerable to antiepileptic DILI because they present a higher activity of CYP450 enzymes, which leads to an increased formation of hepatotoxic metabolites compared with adults [45,46].

Nicoletti et al. reported that 8 of 9 African American patients who developed phenytoin-induced hepatotoxicity carried this allele. These findings underscore the ethnic specificity of *HLA*-linked susceptibility patterns and the importance of population-based pharmacogenetic characterization in preventing severe adverse drug reactions [46]. Supporting evidence comes from Thomas et al., who identified a similar association between HLA-B*53:01 and raltegravir-induced drug reaction with eosinophilia and systemic symptoms (DRESS) [45,47]. This overlap suggests that HLA-B*53:01 may predispose to a shared immunopathogenic mechanism underlying both hepatic and systemic hypersensitivity reactions across structurally and pharmacologically unrelated drugs [45,47]. The recurrence of this allele in multiple immune-mediated toxicities reinforces its potential role as a class I *HLA* risk factor in idiosyncratic, immune-driven adverse drug responses.

### 3.13. Tumor Necrosis Factor-Alpha (TNF-α) Inhibitors

This group of drugs, which includes infliximab, adalimumab, and etanercept, has revolutionized the management of autoimmune and inflammatory diseases, including rheumatoid arthritis, inflammatory bowel disease, and psoriasis. Despite their clinical efficacy, these biologic agents have been increasingly associated with immune-mediated hepatic injury that may present with features resembling AIH. The precise mechanisms underlying these reactions remain unclear, but accumulating evidence suggests that *HLA*-restricted immune responses may predispose certain individuals to hepatotoxicity during anti-TNF therapy [48].

Infliximab-induced DILI has been particularly associated with *HLA* class I alleles, most notably HLA-B*39:01, detected in 25% of DILI cases but absent among controls, despite its low frequency of only 2–2.3% in Caucasians [48]. Among severe cases, defined by ALT levels greater than five times the upper limit of normal (ULN), the association was even stronger, with 30% carriage. All patients carrying HLA-B*39:01 also possessed HLA-C*12:03, suggesting a haplotypic association. Although HLA-C*12:03 was observed in a few control subjects, none carried both alleles, suggesting a specific *HLA-B39:01–C12:03* haplotype that may confer a distinct risk for infliximab-related hepatotoxicity [48]. This haplotype, present in only ~1.3% of the general Caucasian population, was identified in 25% of DILI cases, representing a striking overrepresentation and supporting an immune-mediated mechanism underlying anti-TNF-associated liver injury [48].

### 3.14. Herbal and Dietary Supplements (HDS)

Although conventional pharmaceuticals account for most cases of idiosyncratic DILI, similar immune-mediated mechanisms have been increasingly recognized in reactions triggered by herbal and dietary supplements (HDS). Green tea extract–related hepatotoxicity represents one of the most clearly defined examples of *HLA*-associated susceptibility [48]. Hoofnagle et al. demonstrated a strong association between green tea–induced liver injury and the HLA-B*35:01 allele, which parallels the class I-mediated immune responses observed with certain drugs, such as trimethoprim–sulfamethoxazole, phenytoin, and allopurinol [49]. While HLA-B*35:01 occurs in only 5–15% of the U.S. population, it was present in 72% of patients with confirmed green tea–related hepatotoxicity and in over 90% of highly probable cases. Carriers typically exhibited hepatocellular injury with rash or fever and moderate to severe disease accompanied by jaundice, closely resembling immune-mediated DILI phenotypes observed with pharmacological agents. In contrast, non-carriers showed atypical or chronic patterns of injury. Notably, the causality assessment in this study was performed before chemical and genetic analyses, suggesting that *HLA*-B*35:01 may serve as a more objective and reproducible biomarker for causality than clinical judgment alone [49]. These findings suggest that herbal supplements and drugs can share common *HLA*-restricted immune pathways leading to hepatocellular injury. Thus, HLA-B*35:01 represents not only a risk marker for green tea–induced hepatotoxicity but also part of a broader spectrum of class I *HLA* alleles, including HLA-B*58:01, HLA-B*53:01, and HLA-A*31:01, that mediate drug- and supplement-induced immune liver injury [48,49].

**Table 1:** Evidence on *HLA* association with DILI.

Drug	*HLA*	References
NSAID (diclofenac, ibuprofen)	HLA-DRB1*04:03 HLA-DQA1*03:01 HLA-A*02:01 (independent protective effect) *HLA*-B*35:03l	Bonkovsky, H.L. et al. [21]
Minocycline	HLA-B*35:02	Urban, T.J. et al. [25]
Nitrofurantoin	HLA-DRB1*11:04 HLA-A*01:01-B*08:01-C*07:01 (liver injury following short-term nitrofurantoin therapy)	Chalasani, N. et al. [26]
Flucloxacillin	HLA-B*57:01 HLA-B*57:03	Nicoletti, P. et al. [28] Monshi, M.M. et al. [29]
Amoxicillin–Clavulanate	HLA-DRB1*15:01	Chalasani, N. et al. [30]
	HLA-DRB1*15:01–DQB1*06:02 HLA-DRB1*07 (protective effect)	Donaldson, P.T. et al. [31]
	HLA-DRB1*15:01–DRB5*01:01–DQB1*06:02	Hautekeete, M.L. et al. [32]
Trimethoprim–sulfamethoxazole	HLA-B*14:01 HLA-B*35:01 (African American subgroup)	Li, Y.J. et al. [33]
Azithromycin	HLA-DQA1*03:01 HLA-DQA1*03:01–DQB1*03:02 (stronger correlation with AZ-DILI than HLA-DQA1*03:01 alone)	Conlon, C. et al. [34]
Antituberculosis	*HLA-C12:02–B52:01* B*52:01 allele demonstrated a stronger individual association than C*12:02 HLA-DQB1*02:01 HLA-DQA1*01:02 (absence) HLA-DQA1*01:03 HLA-DQA1*03:01	Nicoletti, P. et al. [40]
	HLA-DQB1*05	Chen, R. et al. [41]
Carbamazepine	HLA-A*31:01	Nicoletti, P. et al. [42]
Allopurinol	HLA-B*58:01 HLA-B*53:01 HLA-A*34:02	Fontana, R.J. et al. [43]
Ticlopidine	HLA-A*33:03	Hirata, K. et al. [44]
Aromatic antiepileptic drugs (phenytoin)	HLA-B*53:01	Nicoletti, P. et al. [46]
Raltegravir	HLA-B*53:01	Thomas, M. et al. [47]
Infliximab	HLA-B*39:01–C*12:03	Bruno, C.D. et al. [48]
Green tea extract	HLA-B*35:01	Hoofnagle, J.H. et al. [49]

## 4. Further Challenges

The use of *HLA* in the diagnosis and prognosis of DILI raises challenges that will need to be resolved in the future. Understanding the mechanisms by which *HLA* alleles contribute to DILI and AIH remains incomplete. The frequency of *HLA* alleles varies significantly across different populations, limiting the generalizability of associations identified predominantly in European and Asian populations. Risk alleles identified in one population may not be clinically relevant in other populations. GWASs for DILI are largely conducted in European cohorts, limiting applicability to the global population. Cost-effectiveness studies show that *HLA* testing for most drugs associated with DILI does not meet the economic thresholds required for clinical implementation. One of the most significant limitations of *HLA* testing in DILI is the extremely low positive predictive value (PPV), despite high negative predictive value (NPV); most carriers of risk alleles will not develop hepatotoxicity, making preemptive genetic screening unrewarding for most drugs [50,51].

Future progress will require the development of complementary *HLA* biomarkers (cytokines, chemokines, markers of cellular activation), the implementation of preemptive *HLA* testing in expanded pharmacogenomic panels to improve PPV, standardization of next-generation sequencing (NGS) protocols and international reporting of *HLA* results, and large, ethnically diverse prospective cohorts to validate *HLA* associations. Integration of flow cytometry immunophenotyping to characterize the immune response in DILI will be necessary, as well as the development of standardized functional T lymphocyte tests for individualized risk assessment.

In clinical practice, decision-support algorithms should be developed to integrate *HLA* alongside clinical history, serological markers, and histology to differentiate DILI from AIH [52]. Future approaches will require international collaborative efforts to standardize, validate, and develop integrative, personalized models to overcome the current limitations of isolated *HLA* genotyping.

## 5. Conclusions

The involvement of the *HLA* system in the pathogenesis of DILI represents one of the most significant advances in modern pharmacogenetics. *HLA* molecules govern the interaction between the immune system and drug-derived antigens, determining whether these compounds are recognized as self or non-self. Specific *HLA* variants can therefore promote aberrant T lymphocyte activation, leading to hepatic inflammation, hepatocellular necrosis, and, in severe cases, acute liver failure. Understanding these immunogenetic mechanisms has substantial clinical and preventive relevance, as it enables the identification of individuals with genetic predisposition and the implementation of personalized therapeutic strategies. The integration of *HLA* genotyping into clinical practice could transform drug safety monitoring and prescribing practices, thereby reducing the incidence of severe hepatic adverse reactions. Consequently, the *HLA* system should be regarded not only as a genetic marker of susceptibility but also as a key tool in advancing predictive and precision medicine within hepatology.

## Data Availability

No new data were created or analyzed in this study.

## Abbreviations

AC-DILI	Amoxicillin-Clavulanate-Drug-Induced Liver Injury
AED	Aromatic Antiepileptic Drug
AIH	Autoimmune Hepatitis
ALT	Alanine Aminotransferase
ANA	Antinuclear Autoantibodies
ATLI	Antituberculosis Liver Injury
AZ-DILI	Azithromycin-Induced Drug-Induced Liver Injury
CI	Confidence Interval
DI-AIH	Drug-Induced Autoimmune Hepatitis
DI-AILD	Drug-Induced Autoimmune-Like Liver Disease
DILIN	Drug-Induced Liver Injury Network
DNA	Deoxyribonucleic Acid
DRESS	Drug Reaction with Eosinophilia and Systemic Symptoms
FasL	Fas Ligand
GWAS	Genome-Wide Association Studies
HDS	Herbal and Dietary Supplements
*HLA*	Human Leukocyte Antigen
IFN-γ	Interferon-γ
IM-DILI	Immune-Mediated Drug-Induced Liver Injury
MHC	Major Histocompatibility Complex
NGS	Next Generation Sequencing
NPV	Negative Predictive Value
NSAID	Non-Steroidal Anti-Inflammatory Drug
OR	Odds Ratio
PPV	Positive Predictive Value
RNA	Ribonucleic Acid
TB	Tuberculosis
Th	T helper
TMP–SMX	Trimethoprim-sulfamethoxazole
TNF-α	Tumor Necrosis Factor-alpha
ULN	Upper Limit of Normal
US	United States
